# Montessori intervention for individuals with dementia: feasibility study of a culturally adapted psychosocial intervention in Pakistan (MIRACLE)

**DOI:** 10.1192/bjo.2020.49

**Published:** 2020-07-02

**Authors:** Nasim Chaudhry, Sehrish Tofique, Nusrat Husain, Debbie Couture, Paula Glasgow, Meher Husain, Tayyeba Kiran, Rakhshi Memon, Shela Minhas, Afshan Qureshi, Florene Shuber, Iracema Leroi

**Affiliations:** Pakistan Institute of Living and Learning, Pakistan; Division of Older Adult's Mental Health, Pakistan Institute of Living and Learning, Pakistan; Division of Psychology and Mental Health, University of Manchester, UK; Montessori Teachers College, Canada; Montessori Teachers College, Canada; Manchester Global Foundation, UK; Division of Self Harm and Suicide Prevention, Pakistan Institute of Living and Learning, Pakistan; Manchester Global Foundation, UK; Bright Scholars Montessori, Canada; Department of Qualitative Studies, Pakistan Institute of Living and Learning, Pakistan; Montessori Teachers College, Canada; School of Medicine, Global Brain Health Institute, Trinity College Dublin, Ireland

**Keywords:** Dementia, psychosocial intervention, cultural adaptation, low- and middle-income countries, Montessori intervention

## Abstract

**Background:**

Globally, nearly two-thirds of people with dementia reside in low- and middle-income countries (LMICs), yet research on how to support people with dementia in LMIC settings is sparse, particularly regarding the management of behavioural and psychological symptoms of dementia. Understanding how best to manage these symptoms of dementia with non-specialist approaches in LMICs is critical. One such approach is a non-pharmacological intervention based on the Montessori method.

**Aims:**

To evaluate the feasibility and acceptability of a culturally adapted, group-based Montessori intervention for care home residents with dementia and their study partners, who were paid care workers in Pakistan.

**Method:**

This was a two-stage study: a cultural adaptation of the Montessori intervention and a single-arm, open-label, feasibility and acceptability study of 12 participant dyads. Feasibility and tolerability of the intervention and study procedures were determined through the recruitment rate, adherence to the protocol and acceptance of the intervention. Qualitative interviews were undertaken with the study partners. A pre–post exploratory analysis of ratings of behavioural and psychological symptoms of dementia, functional ability and quality of life were also conducted.

**Results:**

The recruitment and retention rates of people with dementia were acceptable, and the intervention was well tolerated by participant dyads. Findings show a reduction in agitation levels and improvement in mood and interest for the activities.

**Conclusions:**

Feasibility studies of low-cost, easy-to-deliver and culturally adapted interventions are essential in laying the groundwork for subsequent definitive effectiveness and/or implementation trials for dementia in LMICs, where awareness and resources for dementia are limited.

## Global context

Up to 60% people with dementia live in low- and middle-income countries (LMICs)^1^ and in South Asia alone, that number is likely to exceed 9 million by 2030.^2^ Unfortunately, the health and social care infrastructure to support people with dementia and their families is least developed in the regions where the dementia burden is highest. Stigma and poor health literacy regarding dementia are prevalent, contributing to low rates of help-seeking by families, lack of identification of dementia and inadequate support services for people living with dementia and their families.^3^

Pakistan is the world's sixth most populous country, and there are currently 8 million people aged 65 years and older who live with enduring psychological and physical conditions.^4^ This has created a significant challenge for the country. In particular, mental health services specifically for older adults are virtually non-existent and, to date, no population-based study involving people with dementia has been undertaken.^4^ As pointed out by recent guidance, building dementia research capacity and capability in Pakistan and developing and evaluating low-cost, easily accessible interventions for people with dementia and their families or care partners in such low-income health economies is essential to support the development of services.^5^

## Impact of dementia

Dementia can have a significant negative effect on both the person with the diagnosis as well as their family. In particular, the behavioural symptoms and personality changes that often accompany the dementia syndrome, including apathy, aggression, depression and hallucinations, may be associated with significantly worse outcomes in people with dementia, as well as high levels of distress, depression and physical symptoms among family members or other care partners.^4,6^ In countries like Pakistan, the primary intervention for behavioural and psychological symptoms of dementia is pharmacological,^7^ and this type of intervention has a limited evidence-base in the context of dementia and may be associated with significant morbidity and mortality.^8^ Thus, there is a need for psychosocial interventions that are cost-effective and easy to deliver in low-resource countries, several of which have a growing evidence base and are included in the National Institute for Health and Care Excellence guidelines for dementia care.^9^ An important aspect of such non-pharmacological management for behavioural and psychological symptoms of dementia is the group of interventions called psychosocial therapies, which have been endorsed in the World Alzheimer Report of 2011,^10^ but are as yet understudied and rarely offered in LMICs, with the exception of a few notable examples such as culturally adapted versions of cognitive-stimulation therapy.^11^

## Evidence on Montessori intervention

One possible low-cost, accessible psychosocial intervention for behavioural and psychological symptoms of dementia is the Montessori approach. The Montessori method is a system of teaching developed by Maria Montessori based on principles to promote learning and independence in children. The teaching activities are designed to help the child to use their senses for learning social, functional and cognitive skills.^12^ In the context of dementia care, this method has been tried in a research setting in people with dementia and found to be moderately effective in improving certain behavioural outcomes such as eating behaviours, agitation and mood.^13^ This intervention is based on designing and offering activities that take into consideration the interests, needs, past experiences and preferences of the group or participants. It has been reported that participants demonstrated positive engagement with the intervention, suggesting that this may be a promising form of support to investigate and improve the care of people with dementia.^13^

The overall aim of the project was to determine whether a culturally adapted, Montessori method–based, group psychosocial intervention for people with dementia in an LMIC setting is feasible and acceptable for further evaluation in a large-scale, fully powered, randomised controlled trial of clinical- and cost-effectiveness and/or implementation. The specific objectives were: (1) to culturally adapt and refine the Montessori method activities for people with dementia in Pakistan; (2) to determine the feasibility of conducting the research in Pakistan, including scoping of the recruitment/referral pathway; (3) to determine the acceptability of this culturally adapted, group-based intervention for people with dementia in Pakistan; (4) and to set up a participant and public involvement (PPI) research group, contributing to capacity and capability for dementia research in an LMIC setting.

## Method

This study (Clinicaltrials.gov registration number: NCT03491774) received a favourable opinion from the National Bio Ethics Committee of Pakistan (reference number 4-87/NBC-290/17/). Montessori InteRvention for individuAls with dementia: A feasibility study of a Culturally adapted psychosociaL intErvention in Pakistan (MIRACLE) was a two-stage study involving cultural adaptation of the Montessori intervention for people with dementia (objective 1); and a single-arm, open-label, feasibility and acceptability study of the adapted intervention in 12 participant dyads (people with dementia) living in residential care, and their study partners, who were paid care workers employed by a residential care home for older adults with physical and/or cognitive disorders (objectives 2 and 3). We addressed objective 4 throughout both phases of the study.

### Study setting

The study was conducted at Darul Sakoun (A Home of Peace and Love), which is a residential care home for older people in Karachi, Pakistan, from June 2018 to November 2018.

### Sample size

Feasibility studies generally do not require formal sample size calculations.^14^ We included 12 dyads, i.e. 24 participants (divided into two balanced groups for ease of intervention delivery), which we considered sufficient to determine the feasibility of delivering this intervention in a Pakistani context, as well as the acceptability and tolerability of the intervention.

### Study participants

#### Participants with dementia: inclusion criteria

Care home residents were invited to participate in the study if they were aged 60 years or older, and had a diagnosis of one of the common forms of dementia with moderate cognitive impairment stage (score of 10–17 on the Montreal Cognitive Assessment (MoCA)).^15^ The subtypes of dementia included were Alzheimer's disease, vascular dementia and ‘mixed dementia’, diagnosed by a local clinician with expertise in dementia, and according to standard clinical diagnostic criteria (i.e. consistent with ‘major neurocognitive disorder’ as per DSM-5).^16^ All participants were able to communicate in English or Urdu, had the capacity to provide informed consent and had a study partner from the care home staff willing to join them for a 60-min Montessori intervention group session twice a week. MIRACLE researchers were trained to ascertain participants’ capacity to consent to the study, assisted by a checklist of criteria, based on the four pillars of capacity (understanding, retaining, weighing the information given by the researcher and communicating their decision regarding participation). Finally, although our interest in offering the Montessori method to people with dementia related to management of behavioural and psychological symptoms of dementia, we decided not to include these symptoms in the inclusion criteria because this was a feasibility study of the adapted intervention and study design and conduct, rather than an evaluation of intervention effectiveness.

#### Participants with dementia: exclusion criteria

Care home residents were not included in the study if they had advanced stage dementia or a mental or physical illness, including sensory impairment, severe enough to preclude them from safely participating in the study, as determined by the principal investigator. Additionally, individuals unable to provide informed consent for the study were not included.

#### Study partner criteria

Paid care workers from the care home where the study took place were invited to become study partners for the people with dementia and to engage in the Montessori intervention group sessions, provided they regularly cared for residents with dementia. In Pakistan, people who live in care homes are most often there because they either have no family or have been abandoned by their family. Thus, in such settings it is not possible to have family members act as study partners. However, in general, care workers form close bonds with residents and are thus able to provide meaningful input into an intervention such as the Montessori method.

### Intervention

The Montessori intervention for dementia was originally developed by Cameron Camp as a manual assisted group therapy based on the principles of teaching developed for children by Maria Montessori.^12^ For the purposes of MIRACLE, we used the manual *Montessori Based Activities for Dementia Volume 2*,^17^ with permission. This manual contains various activities for individuals and groups, including intergenerational activities, and participant-based activities for rehabilitation. It provides step-by-step instructions for creating and conducting each activity so that people with dementia have a feeling of achievement and reward through the accomplishment of these activities.^17^ The activities represent five domains of functioning: cognitive stimulation, life skills, movement and fitness, sensory stimulation and socialisation. The manual, although very adaptable and designed to deliver a tailored intervention, was not entirely suitable for people with dementia and their care workers in Pakistan, and thus required cultural adaptation as outlined below. Following adaptation, each Montessori intervention group activity session lasted up to 60 min and was delivered twice weekly for 12 weeks. During the sessions, different activities were conducted as per participants’ needs, ability, interests and preferences, guided by the culturally adapted study manual.

#### Stage 1: cultural adaptation of the intervention

Adopting an intervention developed elsewhere into a context with a markedly different language and socioeconomic and cultural context requires adaptation before evaluation.^18^ Cultural adaptation enhances the appropriateness, uptake and chance for subsequent ‘scale-up’ of the intervention.^19^ Thus, to adapt the original Montessori method therapy manual (with permission) to the context of a care home in Pakistan, we followed a modified cultural adaptation approach, based on a more complex framework of adaptations outlined by Barrera and Castro.^20^ According to this framework, cultural adaptation should involve four phases, namely information gathering, preliminary adaptation design, preliminary adaptation testing and adaptation refinement. In our study, the information-gathering stage involved a scoping review of the literature for existing psychosocial interventions that might be suitable to a low-resource setting, were potentially adaptable and could be tailored, as well as appropriate to the context of Pakistan. Subsequently, for the preliminary adaptation phase, we convened a PPI discussion group in our stage 1. This involved a group of volunteers, including care home residents, care providers and healthcare professionals. During the discussion, a summary of the Montessori intervention activities was presented by a group facilitator, followed by guided discussion focused on the application and acceptability of activities, including specific questions regarding physical limitations, fluctuating level of alertness and ability to promote self-management; and care workers’ ability to integrate activities into their daily routine. Feedback from the PPI group thus guided timing and frequency of the groups, the need for study partner support and how the care workers were able to take on the role of study partners. PPI feedback also fed into adaptation of the content of the intervention materials, including culturally relevant pictures, sounds, sports and stories. Examples include those outlined in Table 1.

**Table 1:** Examples of content adaptation of the Montessori method activities for the Pakistan context

Domain	Activity
Sensory discrimination	In the ‘Mystery bag’, the golf ball was removed as it is not a common game in Pakistan.
History and geography	Pictures of famous buildings of Pakistan were included instead of those from other countries, such as the Eiffel tower.
Social interaction	The American flag puzzle was replaced with the Pakistan Flag puzzle. Pictures of cleaning of cricket kit replaced golf kit. For the ‘sound match’ activity, relevant common animals and birds of Pakistan were inserted.

#### Stage 2: delivery of the adapted intervention

This stage of our study addressed the latter two requirements for cultural adaptation of a psychosocial intervention outlined by Barrera and Castro.^20^ Following the baseline assessment, the researchers met all participants in the first 2 weeks (zero visit) to ascertain in detail their background and preferences for activities, to build rapport and to tailor the activities to be undertaken by the group in the subsequent sessions. From the third week onward, participants were invited to attend the 60-min group sessions 2 days a week for a duration of 12 weeks in a group setting (up to six dyads per group).

### Study procedures

#### Recruitment, screening and outcome assessments

Residents from the Darul Sakoun residential care home for older adults were approached by trained researchers and invited to participate in the study. After obtaining informed consent from those who were eligible and agreed to participate, a baseline assessment was completed, and the adapted intervention was delivered over a 12-week period. A further assessment, repeating the baseline measures, was completed at week 12.

### Outcomes

#### Feasibility of the study procedures

For the purposes of this study, feasibility was operationalised through the question, ‘Can it work?’^21^ and the parameters to evaluate this included the recruitment rate (the number of dyads referred, proportion of those who consented out of all eligible people with dementia referred from recruitment sites) and the attrition rate (the number of dyads withdrawn/number consented). We also evaluated whether administering the baseline and outcome exploratory assessment battery is feasible (i.e. Can validated Urdu-language outcomes measures be sourced and administered?) and whether research staff can appropriately administer the assessment battery. Aspects such as randomisation and blinding procedures and the ability to undertake a sample size calculation based on the feasibility data were not considered because this was an open-label study.

#### Feasibility of the intervention components and delivery

This was assessed by ascertaining whether the intervention was delivered, received and enacted as intended. We evaluated session duration and feedback about administration and suitability of the intervention components for each session. Data were captured from the researchers delivering the sessions through a detailed log for each session which rated key aspects on a Likert-type scale.^1–5^

#### Acceptability and tolerability of the intervention

Acceptability was defined as the extent to which the participants delivering or receiving the intervention considered it to be appropriate.^22^ Tolerability was defined as the ability to endure the intervention.^23^ These aspects were captured in the following way:

- Patient satisfaction with the intervention was operationalised by a measure of the total duration and frequency of the sessions recorded by the researcher after each session; patient feedback on the assessment and intervention, recorded through interventionist session logs; a patient satisfaction rating scale and qualitative semistructured interviews with patients following the final session.
- Study partner satisfaction with the intervention was measured by ratings (in each session) of whether the patient was interested, motivated, gained a sense of achievement, took the initiative and displayed emotional responses. This rating was completed by using an in-house five-point Likert-type scale (one indicating strongly disagree and five indicating strongly agree).

#### Exploratory pre–post evaluation

We evaluated a possible signal of effectiveness on key dementia outcomes, such as behavioural and psychological symptoms of dementia, cognition and effect of the intervention on caregiver burden, by cautiously examining any change in assessment rating scales from baseline to end of therapy. Descriptive statistical analysis (mean, frequency, s.d., percentage) was done with SPSS version 22 for Windows.^24^ The patient assessment rating scales included validated Urdu-language versions (or translation) of the following instruments, administered by a trained researcher.

- MoCA: This scale assesses different cognitive domains: attention and concentration, executive functions, memory, language, visuo-constructional skills, conceptual thinking, calculation and orientation. A score of 23 indicates clinically significant cognitive impairment.^25^
- Quality of Life Assessment in Dementia (DEMQOL): This is a 28-item self-report questionnaire for people with dementia. It enquires about feelings, memory and everyday life of people with dementia.^26^
- Geriatric Depression Scale 15-item (GDS-15): This is a self-rated scale of 15 items on which a score of >5 is suggestive of depression and a score of ≥10 is indicative of depression.^27^
- Cohen-Mansfield Agitation Inventory: This scale is used to assess the frequency of agitated behaviours in people with dementia. It consists of 29 items, each rated on a seven-point frequency scale. A higher score indicates a higher level of agitation.^28^
- Disability Assessment for Dementia (DAD): This scale includes questions regarding activities of daily living, instrumental activities of daily living, leisure activities, initiation, planning and organisation, and performance. Higher scores represent less disability in activities of daily living, whereas lower scores indicate more dysfunction. Each item can be scored as follows: 1 point indicates yes, 0 points indicates no and N/A indicates non-applicable.^29^

### Qualitative investigation

Semistructured qualitative interviews were completed at the end of the intervention with five care workers who attended the Montessori intervention sessions. Interviewers used a semistructured topic guide to gather evidence regarding objectives of the feasibility study. All interviews were conducted and transcribed verbatim in Urdu and translated into English to be reported in the manuscripts and then back-translated for assurance of the accuracy of translation. Framework analysis was done to analyse the data, including familiarisation, indexing, charting, mapping and interpretation.^30^ During the initial familiarisation stage, transcripts and fields notes were read by one researcher (S.T.) several times to fully immerse themselves in the data. Then, key themes were identified and a draft theoretical framework was developed. To draft the theoretical framework systematically, indexing was done. During the charting process, data were summarised into table developed as per the theoretical framework draft. This step gave a clear overview of the data. In the final phase, tables were reviewed to assist in data interpretation. To maintain credibility and trustworthiness of the data and subsequent findings, the researcher (S.T.) was supervised by a senior psychiatrist (N.C.) and a senior psychologist with expertise in qualitative research methods (T.K.). These two researchers reviewed all transcripts and a sample of the transcripts was discussed in regular meetings. Engagement in discussion and regular reviews by all team members (S.T., N.C. and T.K.) ensured fit of the data to the final analysis, and helped to minimise bias.^30^

## Results

### Description of the study sample

The mean age was 68.3 (s.d. 5.7) years for the female participants with dementia and 73.1 (s.d. 8.4) years for male participants with dementia. Approximately 25% were married. The mean MoCA score was 13.1 (range 11–17). For study partners, the mean age of females was 33.2 (s.d. 4.3) years and the mean age of males was 25 years (s.d. 0.000). All study partners had at least 2 years of experience as paid care workers. Twelve care workers were initially recruited to participate in the study, although after initial consent, five of them left their jobs (one took maternity leave, three got jobs in some other centres and one moved back to their village). Therefore, screening with care workers was repeated to meet the required number of study partners. Two study partners withdrew midway during the study because of resignations unrelated to the study. No further care workers in the home were available to take part in the study. However, the remaining ten study partners were also taking care of and were familiar with people with dementia whose study partners had left the study, therefore they paired up with the three people with dementia to continue their sessions as participants.

### Feasibility evaluation

#### Recruitment and retention

During the planning phase of the feasibility study, two sites were identified. One potential site was the neurology out-patient department of a public hospital, and the other was a residential care home. Initially there were some difficulties in getting permission for access to the latter. After discussions about the importance of research and building such research capacity in Pakistan, the approval was granted from the residential care home. During the first 2 months of recruitment (from June to July 2018), researchers successfully screened 35 participants from the care home. Of these, 12 people with dementia (nine male and three female) met the eligibility criteria and gave informed consent. None of them withdrew their consent; only one participant had to leave the study as she was moving back into her family home, resulting in a retention rate of 83%. Because of this successful recruitment rate and limited resources, the research team decided not to recruit from the neurology out-patient department.

#### Feasibility of outcome measures

All translated outcome measures were found to be feasible to administer in the Pakistani care home context, despite unfamiliarity of such questions for participants and the relatively low level of formal education of participants relative to their counterparts from high-income countries. However, participants faced difficulty in completing all the outcome rating scales in one session. Thus, to improve engagement and minimise fatigue, assessments were completed in two sittings. The duration of each assessment session took account of the participants’ needs. There was no missing information observed in the data-set.

The research therapist/trainer logs were useful in terms of recording participant dyads’ experiences, as well as trainers’ own experiences of delivering the intervention. Information covered in logs included session number, activity name, activity level (easy or difficult), process of conducting session, time of engagement, participants interest in activity and study partner input in session. The maximum time of engagement reported by the therapist was 25–30 min.

#### Acceptability of the intervention

The adapted version of the intervention was acceptable to all participants including the study partners, as ascertained by the participant's therapy acceptance log, which was completed by people with dementia after each session with the help of a researcher independent to the intervention deliverer. Although the log was self-reported, all participants preferred to complete it with the help of the researcher.

The logs recorded participant feedback on their interest, motivation, initiation, sense of achievement and emotional response (happy, sad, anger, anxious or scared). A total of 89% of participants reported their emotional response as happy. Ratings from the logs show that 90% of participants were interested in completing Montessori intervention activities, 83% were motivated to complete or fully participate in the intervention and 85% reported a sense of achievement at the end of the intervention.

Similar qualitative feedback was received from study partners, which supports the therapy acceptance log feedback: ‘They enjoyed the sessions a lot and sometimes they also asked about you (the interventionist)’ (paid care worker 4).

#### Tolerability of the intervention

There were no adverse events related to the intervention and all except one people with dementia completed all sessions of the study, indicating that the intervention was well tolerated by the participants.

#### Intervention fidelity

The intervention was delivered by a psychologist already trained in Montessori methods (S.T.). Regular supervision from developing the material to intervention delivery was offered by S.M., who is a trained experienced teacher in Montessori methods. S.M. has received training from Dr Cameron Camp in dementia and Montessori method activities and now delivers such training in Canada and Pakistan.

#### Signal of effectiveness

Despite the small sample size and open-label study design, it was important to explore for a possible signal of effectiveness to guide a subsequent controlled trial. We thus examined the difference between pre and post assessment scores on the GDS-15 and the Cohen-Mansfield Agitation Inventory total and subscale scores. We found no significant differences from baseline to end-point at week 12 on any of these scales, including the physical/aggressive subscale, physical/nonaggressive subscale and verbal/nonaggressive subscales. However, we did find a significant pre–post difference on the Cohen-Mansfield Agitation Inventory verbal/aggressive subscale (*P* = 0.005). There was slight improvement on pre–post assessment scores on the initiation domain, performance domain of the DAD (mean difference = 1.4, *P* = 0.34), (mean difference = 2.67, *P* = 0.34) and the DAD total score (MD = 0.90; *P* = 0.54). Similarly, there was a slight improvement from pre–post assessment on DEMQOL (MD = 5.45, *P* = 0.27).

### Qualitative interviews

Five themes emerged from the framework analysis of semistructured interviews. These were experience of working with older adults, views regarding the adapted Montessori intervention, barriers in delivering the adapted Montessori intervention, feedback about the therapist/trainer and suggestions for future studies. Details of the themes and exemplar statements supporting these themes are outlined in Table 2.

**Table 2:** Details of the themes and exemplar statements supported by the qualitative evaluation of five interviews with paid care workers (PCW)

Theme	Details	Exemplar quotation
Experience of working with older adults	Study partners reported mixed experiences with residents, stating that the sessions were overall good, but at times challenging because of fluctuations in mood, behavioural problems, verbal outbursts and health issues.	‘Physical health issues are mostly faced by the caregivers, if the elderly resident has some skin related issues and which is contagious, the caregiver can catch the infection’ (PCW1); ‘Yes, they do have a lot of mood swings. At one moment they are very cooperative, but in the next they become totally opposite, like at one moment they agreed to take a bath, but when they were taken to the washroom, they started beating us. Their behaviour changes so abruptly’ (PCW2).
Views regarding the adapted Montessori intervention	Montessori activities were perceived as unique and interesting as these activities were pictorial and performance-based rather than just observation. Participants took interest in all the activities.	‘The activity that you did animal sound match. They enjoyed a lot’ (PCW3); ‘The activity that you did in which they have to open and close the button was very good and useful as sometimes they have to button up. Although paid care workers help them to get ready’ (PCW3).
Barriers in delivering Montessori intervention	Possible barriers reported by PCWs in delivering this intervention are engagement of staff in routine activities in older adults’ residential homes	‘Many times I had to send your team back without doing any work. I am very sorry for that as sometime our staff are bit stuck in other activities that's why I was unable to get participant to deliver some of your sessions on time’ (PCW2); ‘Every care worker have different responsibilities and schedule due to which it is difficult for all of us to attend something at same time’ (PCW3).
Feedback about the therapist/trainer	According to the study partners, trainers were very cooperative and respectful toward the participants; they highlighted the need of additional trainers during the intervention sessions.	‘If more people come then you will enjoy a lot. As you will be able to engage more residents and able to do things in a better way’ (PCW3); ‘They were all very cooperative. The best thing about you people was, that you were not distant from the residents. You treated them like your family members. You sat with them and performed the activities’ (PCW4).
Suggestions for further studies	To have additional sessions for people with dementia, involve family members so that the activities can also take place at home. They found 1 h for the intervention to be more than enough as people with dementia get exhausted after 1 h. They also suggested adding some outdoor activities to improve socialisation skills.	‘Number of sessions need to be increased because memory impaired patient, whose memory is already fading, would be requiring sessions on daily basis’ (PCW2); ‘More physical activities need be included as in physical activities they get involved easily’ (PCW4).

### Capacity and capability building

Building research capacity and capability in Pakistan, further developing understanding and improving skills in diagnosing and managing people with dementia and in their study partners was a secondary objective of the study to prepare for subsequent definitive research studies. Briefly, the work involved in setting up and orienting the PPI group, training the researchers in study conduct and delivery (including clinical outcome rating scales) and developing the research culture within the care home setting. All these contributed to developing dementia research capacity and capability at an individual and team level, rather than an institutional or system level.

## Discussion

This is one of the first intervention studies for people with dementia in Pakistan, and as such, represents an important step in fulfilling the goals of the ‘Roadmap for developing dementia research in Pakistan’.^5^ Our key finding was that this culturally adapted, low-cost pragmatic intervention was acceptable to and well tolerated by people with dementia and their study partners in a care home setting, and may have a role in reducing behavioural and psychological symptoms of dementia, improving quality of life and some aspects of functional ability. Importantly, we were able to demonstrate that the study could be feasibly undertaken in a residential care home setting that was research-naïve and with paid, minimally trained front-line care workers. Our findings support the need for further work to develop the ‘implementation readiness’ of the intervention and move toward a definitive, fully powered, randomised controlled trial of the intervention, which may combine an effectiveness evaluation with an implementation arm (i.e. a ‘hybrid’ study design).^31^ Models of interventions with an existing strong evidence base that are adapted to local contexts, feasibility tested and then evaluated with implementation methods have been reported.^11^ This would be an important step in ensuring up-scaling of interventions to improve the care provision for people with dementia in LMICs by ensuring that interventions applied in such settings are appropriate to the cultural context. Additionally, it would further the applied dementia research agenda in Pakistan.

The intervention activities and material were carefully adapted to ensure that they were culturally appropriate while still retaining their flexible, pragmatic style and keeping the cost to a minimum. There is now good evidence on the importance of cultural adaptation and its effect on outcomes.^32^ Although our adapted Montessori intervention was feasible and acceptable for both people with dementia and their study partners in a care home setting, certain modifications may still be required to make the intervention suitable to be delivered in home or other settings. This is an important consideration because most of the care for people with dementia in Pakistan is delivered at home in the family context. In Pakistan, there are very few care homes, and for those that do exist, recognition of the presence of dementia in their residents is low.

Obtaining permission to conduct the study in the care home was initially challenging. This was likely because of the low level of understanding within the care home management of the need for and role of research in supporting the development of services. To move the work forward, an important task will be to increase awareness of the need to obtain evidence derived from local research programmes. This task is one of the ten key priorities for dementia research identified by the research roadmap.^5^

Another challenge we faced was adapting the timing of our intervention. Care homes have their own schedule and routine both for residents and care workers,^33^ which can sometimes be difficult to change without the involvement of the centre's manager. This was highlighted by the study partners in qualitative interviews and was the main reason they initially refused to participate in the study. Thus, to obviate this challenge, we worked hard to engage the care manager, as per the recommendations for dementia research–based in care homes outlined by the UK's National Institute of Health Research's ‘Enabling Research in Care Homes’ programme (https://enrich.nihr.ac.uk). Because managers are key in enabling research in care homes, it is essential to engage them from the very beginning and throughout the research programme.

The retention rate of our participants with dementia was beyond our expectations. Only one person withdrew from the study and only because they was returning to their family home. In contrast, retaining study partners (the care workers) proved challenging. In the care home sector in many countries, including Pakistan, the turnover rate of care home workers is high, making it difficult to develop interventions based on their involvement. Moreover, the study partners were at times not available for sessions as they are often busy with core care tasks for other residents. This issue can possibly be addressed by involving family members as there is evidence of positive engagement when family members during their visit did Montessori intervention–based activities.^34,35^

All assessment measures were found to be feasible and easy to administer. However it was observed that it was difficult for people with dementia to respond to all the outcome questionnaires in a single session owing to fatigue, which is common among older adults.^36^ Therefore, the assessments were completed in two sessions rather than one.

The effect of the intervention on a participant's life was explored through both qualitative and quantitative measures. Our quantitative findings showed improvement in participants’ agitation, which was also supported by the qualitative findings from interviews with study partners. This is consistent with a previous study in a high-income country, which found a more positive effect in care home residents with dementia who received a Montessori intervention compared with those who received a control intervention.^37^

The incidence of depression associated with dementia is high and can often lead to impaired functioning.^38^ Our sample size was small, and so we were not expecting an effect on outcomes such as depression. However, study partners reported improvement in residents’ mood and better engagement during the intervention. Previous studies also highlighted the positive effect of the Montessori intervention in functional ability.^37^

Finally, a secondary objective of our study was developing capacity and capability for conducting dementia research in Pakistan, supporting the principles outlined by the ‘Roadmap for developing dementia research in Pakistan’^5^ mentioned above. In areas with low research activity, like in many LMICs, embedding capacity and capability development aspects in existing research projects can be means to add value to the work and foster sustainability and follow-on definitive studies.^5^ Thus, although our capacity and capability development work was limited to the individual and team level, every aspect of the project involved upskilling new researchers and fostering a research culture in a hitherto research-naive setting.

### Strengths and limitations

To our knowledge, this is the first feasibility study of a culturally adapted Montessori Intervention for people with dementia in an LMIC setting. The study was conducted in a well-established older adult care home with paid care partners, where the available services were much better compared with other such homes in Pakistan. Further limitations are the small sample size, the open-label study design and the high attrition rate of the study partners in this study. Staff turnover in care homes is a challenge in both LMICs as well as high-income countries, and this will need to be considered when designing future appropriately powered trials or extending this line of work by including family members or volunteers or trained staff to perform regular activities by following Montessori method principles. Finally, the measurements, although translated into the local language and feasible to administer (as demonstrated in MIRACLE), had not all been fully validated in a Pakistani setting. Practitioners should be careful to only adopt interventions that have been culturally adapted and have undergone appropriate feasibility testing before full-scale definitive testing.

In conclusion, this feasibility study demonstrated that culturally adapted, Montessori method–based activities were well tolerated by the participants. Also, establishing the feasibility and acceptability of low-cost, easy-to-deliver psychosocial interventions is essential in laying the groundwork for successful larger effectiveness or implementation trials in LMICs, where awareness and resources regarding older adults’ mental health may be limited.

## Data Availability

The lead author (N.C.) and project lead (S.T.) had access to the study data. The data that support the findings of this study are available from corresponding author (N.C.) on reasonable request.
